# Median nerve neuropathy in the forearm due to recurrence of anterior wrist ganglion that originates from the scaphotrapezial joint: Case Report

**DOI:** 10.1186/1749-7221-7-1

**Published:** 2012-01-19

**Authors:** Kiyoshi Okada, Junichi Miyake, Toshiyuki Kataoka, Hisao Moritomo, Tsuyoshi Murase, Hideki Yoshikawa

**Affiliations:** 1 Department of Orthopaedics, Osaka University Graduate School of Medicine, 2-2 Yamadaoka, Suita, Osaka 565-0871, Japan; 2 Medical Center for Translational Research, Osaka University Hospital, 2-15 Yamadaoka, Suita, Osaka 565-0871, Japan

## Abstract

**Background:**

Median nerve neuropathy caused by compression from a tumor in the forearm is rare. Cases with anterior wrist ganglion have high recurrence rates despite surgical treatment. Here, we report the recurrence of an anterior wrist ganglion that originated from the Scaphotrapezial joint due to incomplete resection and that caused median nerve neuropathy in the distal forearm.

**Case presentation:**

A 47-year-old right-handed housewife noted the appearance of soft swelling on the volar aspect of her left distal forearm, and local resection surgery was performed twice at another hospital. One year after the last surgery, the swelling reappeared and was associated with numbness and pain in the radial volar aspect of the hand. Magnetic resonance imaging revealed that the multicystic lesion originated from the Scaphotrapezial joint and had expanded beyond the wrist. Exploration of the left median nerve showed that it was compressed by a large ovoid cystic lesion at the distal forearm near the proximal end of the carpal tunnel. We resected the cystic lesion to the Scaphotrapezial joint. Her symptoms disappeared 1 week after surgery, and complications or recurrent symptoms were absent 13 months after surgery.

**Conclusions:**

A typical median nerve compression was caused by incomplete resection of an anterior wrist ganglion, which may have induced widening of the cyst. Cases with anterior wrist ganglion have high recurrence rates and require extra attention in their treatment.

## Background

Anterior wrist ganglion, which mainly originates from the radiocarpal joint, accounts for 15%-20% of wrist ganglions [1,2]. Anterior wrist ganglion show high recurrence rates [3], and we experienced a recurrent ganglion related to incomplete surgical resection. Although some previous reports suggest that carpal tunnel syndrome is occasionally caused by ganglion cysts [4], few reports demonstrate median nerve neuropathy in the forearm due to a ganglion cyst. Furthermore, no reports have suggested that recurrence of anterior wrist ganglion that originated from the Scaphotrapezial joint caused median nerve neuropathy in the forearm. Here, we report a rare case of anterior wrist ganglion recurrence, which originated from the Scaphotrapezial joint, which caused median nerve neuropathy in the forearm.

## Case presentation

**Figure 1:**
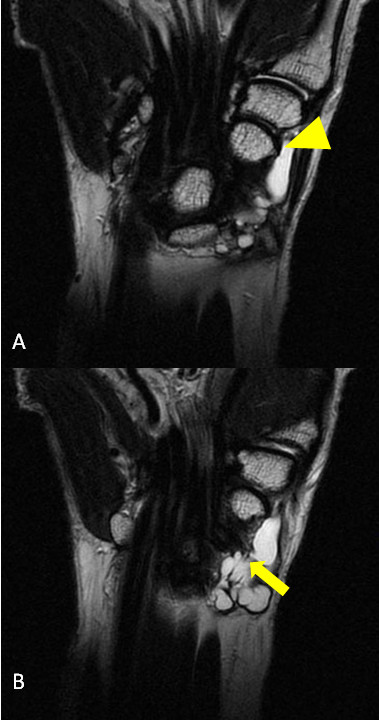
Magnetic resonance imaging results. (**A, B**) T2-weighted fat-suppressed magnetic resonance image showing origination of the multicystic lesion (arrow) from the Scaphotrapezial joint (A; arrow head) and expansion to the distal forearm beyond the wrist (B).

A 47-year-old right-handed housewife noted a soft swelling on the volar aspect of her left distal forearm 3 years ago. She was diagnosed with a ganglion, and local resection surgery was performed twice at another hospital. Similar methods were used to perform both operations, which involved intralesional resections of the cyst with 1-cm skin incisions of the volar aspect of the forearm without following the stalk of the cyst. However, 1 year after the last surgery, the swelling reappeared and was associated with numbness and pain in the radial volar aspect of the hand, thumb, index finger, and middle finger. She came to our hospital and a physical examination revealed decreased sensitivity of the left median nerve area associated with a positive Tinel's sign on the volar aspect of the distal forearm. The patient had no evidence of polyneuropathy, vasculitis, tuberculosis, hypertrophic tenosynovitis, sarcoidosis, gout, or other systemic disorders. She did not give history of previous trauma except for the surgeries at the other hospital, and her wrist joint showed no signs of carpometacarpal arthrosis or instability. The nerve conduction velocity (44.2 m/s) and terminal latency (5.94 ms) of the left median nerve were prolonged and delayed respectively, compared with those of the contralateral nerve (58.0 m/s, 4.12 ms). A radiograph of her left forearm showed no space-occupying lesions around the median nerve and carpal tunnel, and significant osteoarthritic changes of the carpal bones were not observed. Magnetic resonance imaging revealed that the multi-cystic lesion originated from the Scaphotrapezial joint and had expanded beyond the wrist (Figure 1).

**Figure 2:**
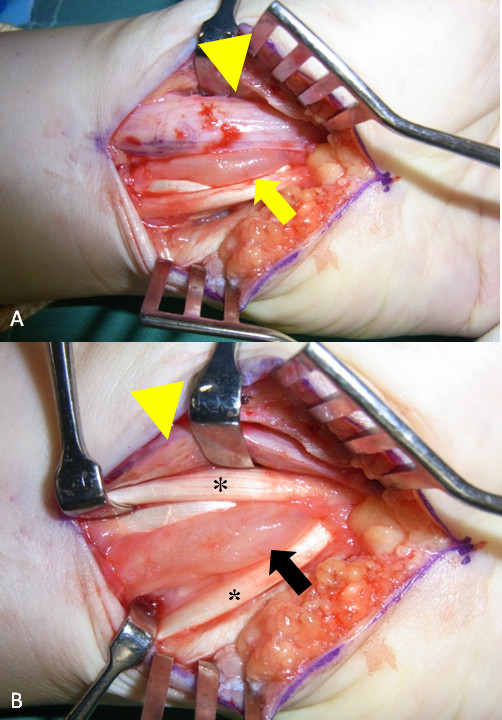
Intraoperative view of the distal forearm showing (A) the median nerve (arrow head) compressed by the large ovoid cyst (arrow) and (B) the large ovoid cyst expanded to the volar of the forearm passing between the flexor carpi radialis (*) and flexor digitorum superficialis (*).

We decided to perform surgery. Exploration of the left median nerve showed that it was compressed by a large ovoid cystic lesion at the distal forearm near the proximal end of the carpal tunnel (Figure 2). We resected the cystic lesion, following the stalk of the cyst to the Scaphotrapezial joint with elongation of the incision, and released the carpal tunnel simultaneously. Nerve compression was observed only around the cyst in the distal forearm and not in the carpal tunnel. Her symptoms had disappeared 1 week after surgery, and pathological examination of the resected cystic lesion showed an appearance consistent with a ganglion. On the basis of these findings, we diagnosed median nerve neuropathy in the forearm due to recurrence of anterior wrist ganglion originated from the Scaphotrapezial joint. Complications or recurrent symptoms were absent 13 months after surgery.

## Discussion

Carpal tunnel syndrome is a common compressive neuropathy of the upper extremities and occasionally caused by ganglion cysts [5]. Kerrigan et al. reviewed ganglion cysts and carpal tunnel syndrome [4] and found some recent reports of carpal tunnel syndrome caused by space-occupying lesions associated with ganglions [6-8]. Although some of these reports have featured typical neuropathies caused by ganglions [9], none clearly demonstrated a relationship between recurrence of anterior wrist ganglion and median nerve neuropathy in the forearm. Here, we describe a case of recurrent anterior ganglion that originated from the Scaphotrapezial joint and caused compression of the median nerve in the distal forearm.

Anterior wrist ganglion is a common soft-tissue tumor in the wrist and may originate from the radioscaphoid or scapholunate joint [2,3]. Ganglions that originate from the Scaphotrapezial joint are less frequent (33%; 35/104) than those that originate from the Radioscaphoid joints (65%; 68/104), and there is no report of such ganglions that have expanded to the forearm and compressed the median nerve. This case showed that a ganglion originating from the Scaphotrapezial joint can expand to the forearm. Cases with anterior wrist ganglion have high recurrence rates. The recurrence rate for cases treated with aspiration is 83% (20/24) and for those treated by surgical resection is 20% (12/60) [2]. In the present case of anterior wrist ganglion recurrence in a 47-year-old woman, the origin of the cyst was not explored during the earlier local resections. Because of the high recurrence rate, it has been suggested that greater attention should be given to determining the origin of the anterior wrist ganglion by careful dissection of the stalk [2]. In addition, it has been reported that recurrent ganglion cysts have the potential to cause severe neuropathy [10]. Thus, on the basis of these previous reports, we believe that the previous surgeries may have been incomplete, which may have induced expansion of the ganglion and caused median nerve neuropathy by compression. We performed carpal tunnel release simultaneously to observe the median nerve in the carpal tunnel. It remains to be seen whether carpal tunnel release may contribute to recovery in this case because there were no indications of median nerve compression in the carpal tunnel.

On the basis of the findings in this case and those in the previous reports, we suggest that primary total resection of the anterior wrist ganglion with a wide incision to identify structures at risk, such as the radial artery, and allow sufficient exposure to identify the stalk of the ganglion following it down to its capsular attachment. In addition, preoperative detection of the origin of the wrist ganglion by MRI or ultrasound is useful to avoid incomplete surgery. The necessity of carpal tunnel release when the median nerve is compressed in the forearm is controversial, but we believe that carpal tunnel release contributes in special cases where the ganglion expands beyond the wrist and may compress the median nerve in the forearm.

## Conclusions

Cases with anterior wrist ganglion have high recurrence rates and require extra attention during their treatment. This case suggests that anterior wrist ganglion should be completely excised during primary surgery. Incomplete resection may induce widening of the cyst and cause several disorders including median nerve compression.

## Consent

Written informed consent was obtained from the patient for publication of this case report and any accompanying images. A copy of the written consent is available for review by the editor-in-chief of this journal.

## Competing interests

The authors declare that they have no competing interests.

## Authors' contributions

KO performed the surgery, performed all pertinent literature reviews on the subject, and drafted the manuscript. JM assisted KO in the surgery. TK, HM, TM, and HY helped to draft the manuscript. All authors read and approved the final manuscript.
